# Effects of Immersive Virtual Reality on Physical Function, Fall-Related Outcomes, Fatigue, and Quality of Life in Older Adults: A Randomized Controlled Trial

**DOI:** 10.3390/healthcare13151800

**Published:** 2025-07-24

**Authors:** Damla Parmak, Ender Angın, Gozde Iyigun

**Affiliations:** Physiotherapy and Rehabilitation Department, Faculty of Health Sciences, Eastern Mediterranean University, 99628 Famagusta, North Cyprus, via Mersin 10, Turkey; ender.angin@emu.edu.tr (E.A.); gozde.iyigun@emu.edu.tr (G.I.)

**Keywords:** exercise therapy, virtual reality, physical function, risk of falling, fear of falling, fatigue, quality of life, older adults

## Abstract

**Background/Objectives:** This study aimed to evaluate the impact of an immersive virtual reality (IVR) program on balance, physical fitness, risk of falling, fear of falling, fatigue, and quality of life in older adults compared with an active control group (ACG). **Methods**: A total of 44 older adults were randomly assigned to either the IVR group (n = 22) or the ACG (n = 22) for an 8-week period. The IVR group participated in 35-min immersive virtual reality sessions three times a week, whereas the ACG followed a home-based traditional exercise program. Evaluations were conducted both before and after the intervention period. **Results**: Compared with the ACG, the participants in the IVR group demonstrated significant improvements in balance, upper and lower extremity strength, lower extremity flexibility, fatigue levels, and specific aspects of quality of life such as autonomy and social participation. Treatment satisfaction was also higher in the IVR group. **Conclusions**: An 8-week immersive virtual reality intervention was effective in improving physical function, reducing fatigue, and enhancing specific domains of quality of life among older adults.

## 1. Introduction

The World Health Organization (WHO) defines old age as individuals aged 65 years or older [1]. The aging of the population is undergoing dynamic changes as a result of the increase in life expectancy in all countries worldwide [2,3]. The number of individuals aged 65 and older is projected to reach 2.1 billion by 2050, while the population aged 80 and older is expected to reach 426 million between 2020 and 2050 [4,5]. In light of these developments, maintaining functional independence among older adults has become increasingly important in this rapidly changing demographic landscape, in parallel with the changing health needs associated with the natural aging process [6,7]. Physical fitness parameters are important for maintaining functional independence and overall health in older adults [8]. Advancing age is associated with declines in muscle strength, flexibility, aerobic endurance, and balance—core components of physical fitness—which collectively contribute to a reduction in overall physical fitness [9]. The deterioration of associated components results in balance difficulties in older adults, hence heightening the risk of falls [10]. Maintaining balance is a complex motor skill that requires the integration of sensory inputs, movement planning, and execution [11,12]. Approximately 30% of individuals aged 65 and older and 50% of individuals aged 80 and older experience at least one fall per year, and one-third of those who fall experience recurrent falls [13,14,15]. Fear of falling is common among older adults following a fall and is associated with restricted daily functioning, decreased social engagement, lower physical activity levels, and a reduced overall quality of life [16,17,18,19]. As people age, there is a marked decline in their overall quality of life. This decline is particularly concerning during the advanced stages of life, often referred to as old age.

Regular physical activity plays an important role in maintaining and improving health, extending the average life expectancy, and reducing all-cause mortality rates [20,21]. A comprehensive range of physical activities, including aerobic exercise, strength training, balance exercises, flexibility exercises, yoga, tai chi, and Pilates, is recommended for older adults. However, the tendency to adopt a sedentary lifestyle increases with age; various barriers such as fatigue and transportation issues further complicate this process [22,23,24,25]. Additionally, the inadequacy of exercise interventions for the elderly, combined with their perception of traditional exercise interventions as boring, can lead to a lack of motivation and compliance issues [26,27,28,29]. As a result, virtual reality has emerged as an important digital technology for enhancing exercise programs, reducing boredom, and increasing motivation.

Virtual reality is defined as an interactive, three-dimensional technological environment created using hardware and software that can simultaneously address multiple sensory inputs and provide individuals with the feeling of being in a real environment through simulation support [30]. According to the concept of immersion, which is used to express the level of interaction between an individual and the virtual world, virtual reality is classified as follows: non-immersive virtual reality is a type of virtual reality in which individuals interact using a controller or computer mouse, and a virtual image is projected onto a computer or television screen [30,31,32]. Semi-immersive virtual reality is facilitated by larger screens, which generate a more intricate virtual environment with sophisticated graphics [30,31,32]. Immersive virtual reality employs high-quality graphics to present users with a 360° view of a virtual environment through a head-mounted display (HMD) [30,31,32]. A multitude of studies have demonstrated the multifaceted benefits of virtual reality (VR) interventions, emphasizing their positive impact on the physical, cognitive, and psychological well-being of older adults. The accessibility, entertainment value, and capacity to facilitate social interaction of VR have been identified as key factors contributing to its efficacy [33,34,35,36]. In particular, the motivational and participatory benefits of virtual reality have been emphasized [37,38]. Virtual reality applications are considered safe for older adults due to their ease of use and minimal discomfort [39]. Furthermore, virtual reality interventions have been observed to be more engaging than traditional methods due to their visual and auditory feedback and the gamified presentation of repetitive tasks [33]. This is of particular importance in the current digital age, where digital technologies such as virtual reality can be used to encourage older adults to participate in physical activity programs. Research has demonstrated that virtual reality interventions enhance parameters such as balance performance and lower extremity muscle strength in older adults, providing substantial support for rehabilitation [40,41]. Moreover, it has been documented that virtual reality interventions hold promise in enhancing social and emotional well-being, and consequently, the quality of life in older adults [42,43]. A review of the existing literature, including virtual reality interventions, indicates a predominance of research centered on elderly adults suffering from neurological and orthopedic conditions. The evaluated outcomes of these studies are predominantly constrained to single-dimensional physical outcomes, and the utilization of non-immersive virtual reality systems is prevalent. Therefore, the objective of this study was to make a meaningful contribution to the existing literature by adopting a comprehensive approach that simultaneously evaluates interconnected physical and psychosocial parameters in older adults. The primary aim of the study was to examine the effectiveness of an immersive virtual reality intervention in improving balance performance compared to an active control among community-dwelling older individuals. Secondary objectives included evaluating the effects of immersive virtual reality on physical fitness, risk of falling, fear of falling, fatigue, and health-related quality of life. The findings aim to inform the integration of innovative, technology-enhanced strategies into physiotherapy practice to support functional independence and well-being in the aging population.

## 2. Materials and Methods

### 2.1. Study Design

The study was conducted in accordance with the Declaration of Helsinki, and received ethical approval from the Board of Scientific Research and Publications of the Eastern Mediterranean University (SBF00-2024-0128). The trial was also registered at ClinicalTrials.gov (identifier: NCT07007026). It employed a randomized controlled trial, repeated measures, and a quantitative approach for two groups: an immersive virtual reality (IVR) group and an active control group (ACG). The duration of the interventions was 8 weeks in total, with 3 sessions per week (24 sessions). All individuals included in the study were evaluated before the intervention and at the end of the intervention eight weeks later. Cognitive status, sociodemographic information, physical fitness, balance, risk of falling, fear of falling, fatigue, quality of life, treatment satisfaction, and virtual reality sickness were evaluated. Block randomization was performed with a block size of 4, and within each block, 2 participants were randomly assigned to the IVR group and 2 participants to the ACG. The randomization sequence was generated by an independent statistician using GraphPad Prism 7.0 software with the aim of ensuring balanced group distribution and allocation concealment. After participant enrollment and baseline assessments were completed, randomization was performed by an independent statistician. The participants were informed that they would receive an exercise intervention but were blinded to whether they were assigned to the immersive virtual reality (IVR) group or active control group (ACG) to prevent performance bias. Due to the nature of the interventions, it was not possible to blind the researcher responsible for delivering the interventions and conducting the assessments. All assessments and interventions were performed by the same physiotherapist (D.P.).

### 2.2. Participants

**Figure 1:**
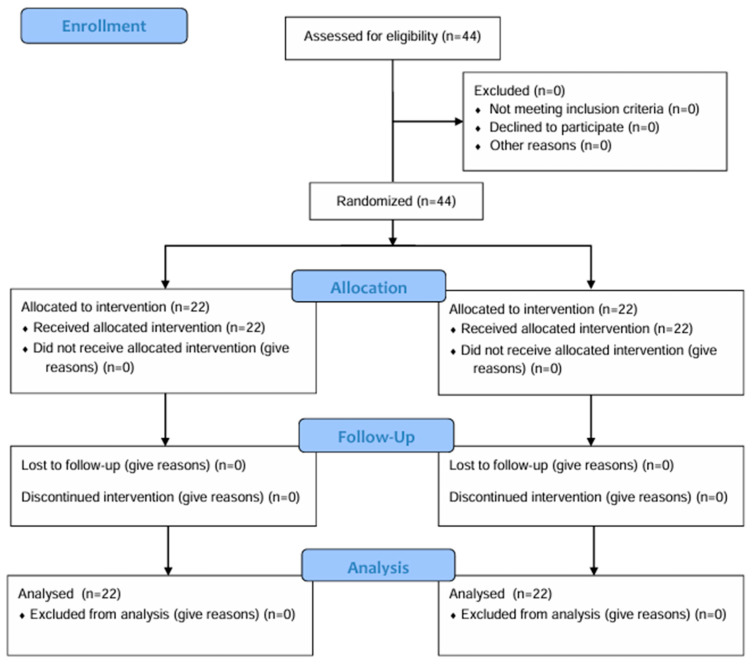
CONSORT 2010 flow diagram of study design.

This study included individuals aged 65 or over. The sample size was calculated using the G*Power (version 3.1.9.4) computer program. The rationale for the effect size was based on the findings of a previous randomized controlled trial by Zahedian-Nasab et al., which investigated the effects of virtual reality exercises on balance and falls in older adults [44]. The previous study found that virtual reality exercise improved balance and fear of falling in older adults. Consequently, the effect size was determined using this study by Zahedian-Nasab et al. The Cohen’s d effect size was calculated to be 1.12. Statistical power analysis calculations suggested 18 subjects per group (α = 0.05, β = 0.20, 95% power) [44]. Considering that participants may leave the study for any reason during the eight-week training period, the sample size was increased by 20% to include a total of 44 participants, with 22 participants in each group. The protocol for this study is illustrated in the CONSORT flowchart in Figure 1, and Table 1 summarizes the sociodemographic characteristics of the sample, while Table 2 summarizes the anthropometric characteristics.

The inclusion criteria were as follows: (i) individuals aged 65 or over; (ii) a Mini-Mental State Test Score of 24 or over; (iii) the ability to walk independently without using an assistive device; and (iv) agreement to participate in the study voluntarily. The exclusion criteria were as follows: (i) a diagnosis of neurological diseases (stroke, Parkinson’s, or multiple sclerosis); (ii) a diagnosis of cardiovascular disease (heart failure, endocarditis, myocarditis, or cardiac arrhythmias); (iii) a diagnosis of psychiatric or cognitive disorders; (iv) surgical operation in the last 6 months; (v) having a visual impairment that makes it impossible to see virtual reality images; and (vi) a diagnosis of vertigo, epilepsy, or cancer. Before the intervention, the study’s purpose and the exercise programs’ content were explained to the participants in detail, and they signed a written informed consent form.

### 2.3. Assessments

The primary outcome of this study was balance. Secondary outcomes included physical fitness, risk of falling, fear of falling, fatigue, quality of life, treatment satisfaction, and virtual reality sickness. These outcomes are detailed in the subsections below.

#### 2.3.1. Cognitive Status

The Mini-Mental State Test (MMST) was used to assess cognitive status [45]. The Mini-Mental State Examination Test consists of five domains, namely, orientation (10 points), recording memory (3 points), attention and calculation (5 points), recall (3 points), and language (9 points), and it is evaluated using a total of 30 points. A score of less than 24 indicates cognitive impairment. A range of 24–30 points is considered normal, 18–23 points indicates mild dementia, 12–17 points indicates moderate dementia, and less than 12 points indicates severe dementia. The MMST was translated into Turkish and its validity and reliability were assessed by Güngen et al., who reported high inter-rate reliability (r = 0.99), a kappa value of 0.92, sensitivity of 91%, and specificity of 95% [46].

#### 2.3.2. Balance

The Fullerton Advanced Balance (FAB-T) scale, which was developed to evaluate multidimensional changes, was used to assess balance [47]. The Fullerton Advanced Balance Test is a test consisting of ten items in total, including standing with feet together and eyes closed, reaching forward by extending the arm to pick up an object (pen) held at shoulder level, turning 360 degrees in the right and left directions, stepping on and over 15 cm steps, tandem walking, standing on one leg, standing on foam with eyes closed, two-foot jumping, walking by turning the head, and reactive postural control. Each item is scored between 0 and 4, and the test has a maximum score of 40. A higher score indicates better balance ability, and a lower score indicates poor balance ability. İyigün et al. conducted a validation and reliability study of the Turkish version of the FAB-T, reporting high inter-rater and intra-rater intraclass correlation coefficients (ICCs) ranging from 0.92 to 0.96. These results confirm the FAB-T as a psychometrically robust instrument for assessing functionally independent older adults [48]. The threshold for demonstrating Minimal Clinically Important Differences (MCIDs) in FAB-T scores in older adults is not specified in the literature; the only MCID identified for patients with lymphedema is 2.33 points [49].

#### 2.3.3. Physical Fitness

The Senior Fitness Test (SFT) was used to evaluate the physical fitness parameters of the individuals [50]. The Senior Fitness Test comprises a total of six functional tests that assess muscular strength, flexibility, aerobic endurance, balance, and agility. A 30-s sit-to-stand test was used to evaluate the muscle strength of the lower extremity, and a 30-s weightlifting test was used to evaluate the muscle strength of the upper extremity. A two-minute step test was used to assess aerobic endurance, which involves using a stopwatch to record the total number of dominant side steps taken correctly for 2 min. The sit-and-reach test was used to evaluate the flexibility of the lower extremity muscles, and the back scratch test was used to evaluate the flexibility of the upper extremities. For the assessment of agility and dynamic balance, an eight-step walk test was used, which involved getting up from a chair on command, walking a distance of 2.44 m as fast as possible, but without running, and sitting back on the chair by turning around the cylinder ahead. The Minimal Clinically Important Difference (MCID) for the sit-to-stand test has been reported as 2 repetitions in patients with hip osteoarthritis [51].

#### 2.3.4. Risk of Falling

The Morse Falls Scale (MFS) was used to assess the risk of falls [52]. It is a practical scale that can categorize patients based on their fall risk. It encompasses a total of six domains: presence of a history of falls, presence of comorbidity, use of assistive devices, intravenous therapy status, transfer/walking, and mental status. In the evaluation, fall risk is determined using the cut-off scores suggested by Morse, with 0–24 points indicating no fall risk, 25–50 points indicating a low fall risk, and 51 points and above indicating a high fall risk. The Turkish validity and reliability study of the MFS was conducted by Yılmaz and Seren. Although the Cronbach’s alpha value was reported as 0.55 in the sample group, it was determined that the scale had a high level of reliability in the study in which it was developed and that there was a significant difference between the patients’ fall risk levels and their falls [53].

#### 2.3.5. Fear of Falling

The International Fall Efficacy Scale (FES-I) was used to assess the fear of falling [54]. It is a scale consisting of a total of 16 items, with each item ranging from 1 to 4 points. The minimum total score is 16, and the maximum total score is 64. A high total score indicates a high fear of falling. The Turkish validity and reliability study of the FES-I was conducted by Ulus and colleagues; the scale is a valid and reliable tool for measuring the fear of falling, with a Cronbach’s alpha of 0.94 [55].

#### 2.3.6. Fatigue

The FACIT Fatigue Scale, which assesses the level of fatigue in the last seven days, was used to evaluate fatigue. It consists of 13 items in total, with each item scored on a scale of 0 to 4. The minimum total score is 0, and the maximum total score is 52. The lower the total score, the higher the individual’s fatigue level [56]. The Turkish validity and reliability study of the FACIT was conducted by Çınar and Yavaş; the scale is a valid and reliable tool for measuring fatigue, with a Cronbach’s alpha of 0.92 [57]. The threshold for demonstrating minimal clinically important differences in FACIT scores in older adults has not been specified in the literature; the only MCID identified for patients with rheumatoid arthritis is 15.9 points [58].

#### 2.3.7. Quality of Life

The World Health Organization Quality of Life Instrument—Older Adults Module (WHOQOL-OLD) comprises 6 sub-domains: sensory functions, autonomy, past, present, and future activities, social participation, dying and death, and closeness, with a total of 24 items [59]. Each item is scored on a scale of 1 to 5, with a minimum of 4 points and a maximum of 20 points possible for each sub-domain. The total score is calculated by summing the score for each sub-domain. The maximum total score that can be obtained in the whole scale is 120, while the possible minimum total score is 24. The higher the total score, the higher the individual’s quality of life. The Turkish validity and reliability study of the WHOQOL-OLD scale was conducted by Eser and colleagues. The scale is a valid and reliable tool for measuring quality of life, with a Cronbach’s alpha of 0.85 [60].

#### 2.3.8. Treatment Satisfaction

The Visual Analog Scale (VAS), consisting of a 100 mm long horizontal line, was used to assess treatment satisfaction. There are two descriptors at the beginning and end of the line indicating extreme satisfaction (not at all satisfied and very satisfied).

#### 2.3.9. Virtual Reality Sickness

The Virtual Reality Sickness Questionnaire (VRSQ) consists of two components, oculomotor and disorientation, with a total of 9 items [61]. The items are scored on a 4-point scale ranging from 0 to 3 (0 = none, 1 = mild, 2 = moderate, and 3 = very). The oculomotor impairment component consists of 4 items: general discomfort, fatigue, eye strain, and difficulty focusing. The disorientation component consisted of 5 items: headache, head fullness, blurred vision, dizziness with eyes closed, and vertigo. Oculomotor, disorientation, and total scores are obtained from the questionnaire. Oculomotor and disorientation scores are calculated by dividing the individual’s component score by the total score obtained (as a percentage). The total score is calculated using the simple average method, where a higher score indicates a higher level of movement disorder. The Turkish validity and reliability study of the VRSQ was conducted by Çetin and colleagues. The study demonstrated that the Turkish version of the VRSQ has moderate internal consistency (Cronbach’s α = 0.674 at 1 min and 0.633 at 10 min post-VR); the α values were 0.786 for the oculomotor component and 0.753 for the disorientation component [62].

### 2.4. Intervention

#### 2.4.1. Immersive Virtual Reality

**Figure 2:**
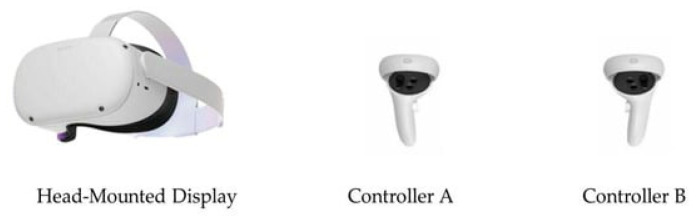
Oculus Meta Quest 2 headset.

In the immersive virtual reality intervention, older adults utilized an Oculus Meta Quest 2 headset (Meta Platforms, Inc., Menlo Park, CA, USA). The Oculus Quest 2 provides an immersive virtual reality experience with a head-mounted display (HMD) and two controllers (Figure 2). It features two built-in speakers, four infrared cameras, an accelerometer, a gyroscope, and two controllers. Its advanced technical specifications include a resolution of 1832 × 1920 pixels, a refresh rate of 90 Hz, a 90° field of view, and head tracking. These features provide a higher level of immersion [63]. Weighing 503 g, the device features a lightweight and comfortable design. Another advantage is that it is a new-generation device that significantly reduces or eliminates potential discomfort associated with virtual reality [64,65]. In addition, the Meta Horizon app on the phone makes screen mirroring easier, allowing task progression to be observed by mirroring the headset screen.

**Figure 3:**
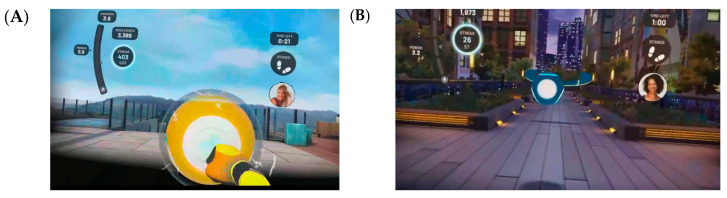
Views of the FIT-XR BOX mode on the Oculus Meta Quest 2 headset. (**A**) First-person view during gameplay. (**B**) Views of different environments in the same exercise mode.

**Figure 4:**
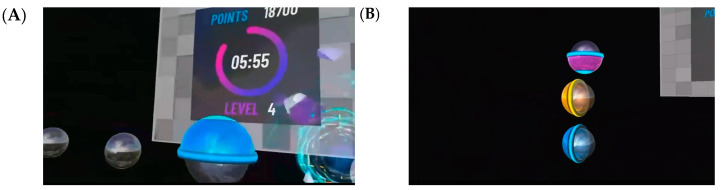
Views of FIT-XR SLAM mode on the Oculus Meta Quest 2 headset. (**A**) First-person view during gameplay. (**B**) Appearance of different balls in the same exercise mode.

The FIT-XR application, available in the Oculus Store, was utilized. It offers a variety of exercise modes and interfaces, including options for indoor and outdoor scenarios. An exhaustive examination of all exercise modes within the application was conducted to enhance upper and lower extremity muscle strength, weight transfer, stepping, trunk control, endurance, coordination, cognitive functions, and the duration and difficulty level of the exercises. The evaluation process involved a meticulous assessment of the suitability of these exercises for elderly individuals. Consequently, the FIT-XR Box and FIT-XR Slam exercise modes were selected for use in the study (Figure 3 and Figure 4). The FIT-XR Box exercise mode was designed to enhance upper extremity movements in various directions, lower extremity movements, trunk movements, weight transfer, speed, and cognitive functions. Within the context of the game, participants are tasked with striking a set of two balls, designated as blue and yellow. This action is guided by the colors of the boxing gloves, which are represented in the virtual environment. The gloves, one blue and one yellow, serve as a visual reference for the players, guiding their physical actions in accordance with the colors they perceive. In the game, the balls projected by individual participants require the player to strike them in a direct and varied manner, as depicted on the balls. Players are required to strike the balls that appear in the game in the direction indicated, with the velocity of their swing determining their score. The hand controllers are analogous to boxing gloves in the game. The game does not require the user to press any buttons. In addition to striking the balls that appear on the screen, the player must also overcome the obstacles that emerge during the game. These obstacles may require the player to move laterally to the left or right, or to assume a squatting position.

The FIT-XR Slam exercise mode has been engineered to promote the development of upper limb movements in multiple directions, weight transfer, stepping, turning, trunk control, speed, and cognitive functions. The game utilizes a set of three colors for the balls: blue, yellow, and pink. In this virtual environment, participants are tasked with striking the blue and yellow balls, which correspond to the colors of the boxing gloves they perceive in their virtual hands. The pink balls, on the other hand, can be struck with either hand. Within the confines of the game, the balls can appear in any of the nine square-meter areas, either as a solitary entity or in multiple forms, with each ball possessing its distinct coloration. The game’s objective is for participants to proceed to the indicated balls and strike them with the hand of the designated color. The objective of the game is to successfully strike each ball within the allotted time, which is contingent upon the difficulty level of the selected game. The player must reach and strike the ball before the designated time expires.

In both of the aforementioned exercise modes, three difficulty modes are present: beginner, intermediate, and advanced. The difficulty levels were determined based on the participants’ progress levels. We would like to note that all the participants experienced the three levels of difficulty in the immersive virtual reality intervention; however, progress and personalization during daily sessions were dynamically adjusted based on multiple factors, including the individual’s performance on that day, their fatigue level, verbal and nonverbal feedback, and their overall health status. This flexible approach optimized participation and safety by ensuring that the program was neither too easy nor overly challenging. Task difficulty, duration, and intensity were adjusted in real time by the physical therapist during the session.

In addition to the exercise modes, it was deemed appropriate to incorporate a warm-up and cool-down program that the individuals could undertake in conjunction with the trainer in the virtual world, with the options available in the application. In the planning stage, particular emphasis was placed on two key factors: the incorporation of rest periods to address the fatigue status of elderly individuals and the sustainability of the exercises. Taking all of these into consideration, an immersive virtual reality intervention session was created:

Warm-up = 3 min;
FIT-XR BOX mode = 5 min;
Rest = 3 min;
FIT-XR BOX mode = 5 min;
Rest = 3 min;
FIT-XR SLAM mode = 5 min;
Rest = 3 min;
FIT-XR SLAM mode = 5 min;
Cool-down = 3 min.
Each session lasted 35 min.

The participants were first provided with general information regarding the training program and the purpose of the virtual reality intervention. The development of weekly session programs was initiated, and days and times that were conducive to the individual’s participation were identified. Before the commencement of the study, the participants were familiarized with the device, and the exercise modes were thoroughly explained. Before initiation of the first session, the exercise modes were meticulously explained verbally and visually by the physiotherapist, and the participant was duly informed. The immersive virtual reality intervention group received virtual reality intervention in the form of individual sessions in their own homes under the supervision of the physical therapist. The intervention program was implemented over a period of eight weeks, with three sessions per week, each lasting 35 min. The participants received continuous supervision and support from the physical therapist throughout all sessions.

#### 2.4.2. Active Control Group

**Figure 5:**
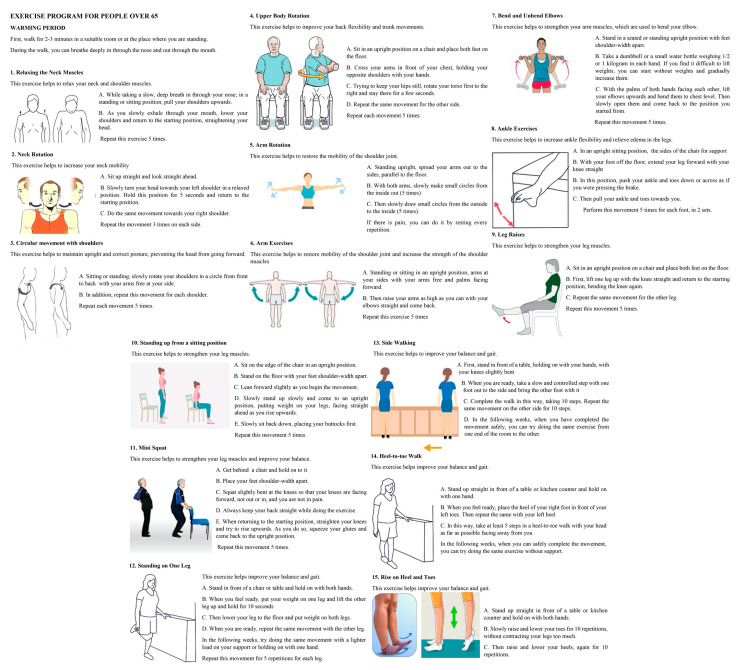
The exercise program developed for the active control group by the physiotherapist.

A physiotherapist with expertise in this field developed a home-based exercise program for the control group, incorporating visual aids and detailed instructions specifically designed to be appropriate for older adults. This program consisted of 24 sessions over 8 weeks, with 3 sessions per week, each lasting approximately 35 min. These sessions were designed to engage the entire body, including the neck, shoulders, trunk, upper extremities, and lower extremities. A warm-up session was also included. The exercise program, comprising a total of 15 exercises, including balance, strengthening, relaxation, and flexibility exercises, was designed in accordance with the outcome measures evaluated in the study. A brochure detailing all exercise specifics (exercise execution, positions, number of repetitions, etc.), accompanied by visual aids, was prepared and distributed to participants (Figure 5). The participants’ compliance was monitored by the physical therapist through weekly phone calls.

The participants in both groups were instructed to refrain from participating in any other training programs without the physiotherapist’s knowledge.

### 2.5. Statistical Analysis

The statistical software package SPSS 27.0 was used for data analysis. Chi-square tests were used to compare the sociodemographic characteristics of participants in the virtual reality intervention and active control groups. The normality of data distribution was assessed using the Shapiro–Wilk test, as well as the skewness and kurtosis coefficients. The findings of this evaluation indicated that the data were normally distributed. To make inter-group comparisons, *t*-tests for independent samples were used to compare the participants’ pre- and post-intervention balance, physical fitness, fall risk, fear of falling, fatigue, and quality of life. To systematically compare changes in balance, physical fitness, fall risk, fear of falling, fatigue, and quality of life between pre- and post- intervention groups, analysis of covariance (ANCOVA) was used to control for baseline differences. The alpha level was set to 0.05. Since only two groups were compared and the number of primary comparisons was limited, corrections for multiple comparisons, such as the Bonferroni adjustment, were deemed unnecessary. An analysis of covariance (ANCOVA) test was performed to examine the effect of treatment based on the groups. A pre-test–post-test design was used, where the dependent variable was the post-test and the pre-test was used as a covariate, not an outcome. A partial eta-squared (η^2^) calculation was used to calculate the effect sizes, and the guidelines proposed by Cohen were used to interpret the results [66]. The effect sizes are interpreted as follows: η^2^ = 0.01 indicates a small effect, η^2^ = 0.06 indicates a medium effect, and η^2^ = 0.14 indicates a large effect [66].

## 3. Results

The results of this study, including both intra-group and inter-group disparities, are comprehensively outlined in the following Table 3, Table 4, Table 5, Table 6, Table 7, Table 8, Table 9 and Table 10.

All participants in the IVR group and the ACG fully participated in the intervention programs, which consisted of three sessions per week for eight weeks, totaling 24 sessions. No participants reported adverse effects during the course of the clinical trial.

## 4. Discussion

The aim of this study was to investigate the effects of an immersive virtual reality intervention program on balance, physical fitness, fall risk, fear of falling, fatigue, and quality of life in elderly individuals compared with an active control group. Compared with the literature, these findings comprise both similarities and differences, and methodological diversity and intervention differences are important factors in interpreting the results.

### 4.1. Balance

When examined in terms of balance, a statistically significant difference was found between the IVR group and the ACG based on the final FAB-T test scores (Table 4). The balance scores obtained by the IVR group in the final test were higher than those obtained by the ACG. The findings of the present study are similar to the results reported in the literature. For example, Rodríguez-Almagro et al. reported in a systematic review comprising 20 studies that virtual reality interventions are superior to traditional balance training in improving balance performance [67]. Bieryla et al. conducted a virtual reality intervention study in elderly individuals using the Xbox three times a week for three weeks. While no significant changes were reported in the control group post-intervention, the virtual reality intervention significantly increased the FAB-T scores [68]. Furthermore, Sadeghi et al. reported better outcomes in balance and functional mobility in elderly men following a virtual reality intervention compared with traditional balance training and a control group [69]. Furthermore, analysis of the differences between the pre-test and post-test scores between the groups in our study showed that the increase in post-test FAB-T scores in the IVR group was higher than that of the ACG. The effect size was also found to be strong (η^2^ = 0.259). The current findings may be attributed to the fact that immersive virtual reality interventions likely have a stronger effect on balance performance by more effectively supporting the involvement of the sensorimotor system through visual, auditory, and motor feedback mechanisms. By enabling participants to respond in real time to environmental stimuli during the intervention, it simultaneously activates the proprioceptive, visual, and vestibular systems that influence balance [70,71]. The support of these multi-system activations with immersive virtual reality interventions that provide rapid and continuous feedback is important for achieving effective balance. For example, Bersotti and colleagues reported that semi-immersive and immersive virtual reality interventions resulted in measurable improvements in mobility and balance performance in older adults, but they also noted that immersive virtual reality interventions may be potentially more effective [72]. The MCID has not yet been reported for FAB-T scores in older adults. However, considering a reference value of 2.33 points [49], the improvement in balance functions in the IVR group (5.73) was well above the MCID limits. The improvement in balance functions in the ACG (2.86) was just above the MCID cutoff. This suggests that IVR interventions may provide a more clinically meaningful change. Ultimately, the immersive virtual reality model we used in this study and the games we selected, which included repetitive movements aimed at improving balance performance, may have supported positive developments in balance performance by encouraging older adults to exert more effort to concentrate during the game, thereby using their muscles more actively. In particular, one of the games we selected, FİT-XR Slam, differs from other virtual reality applications that are performed while sitting or standing in a fixed position, as it requires the elderly individual to actively move around the room in order to complete the tasks. The results obtained in terms of balance performance in elderly individuals support the design of the intervention structure. In this regard, the current findings contribute to the literature by providing a current perspective on immersive virtual reality interventions in different contexts. When designing virtual reality interventions for older adults, the appropriateness of the intervention to the target and the level of interaction should be considered as important factors that can influence the success of the intervention.

### 4.2. Physical Fitness

Comparing the changes between the pre-test and final test scores for the physical fitness parameter revealed that the IVR group showed significant improvements in upper and lower extremity strength and lower extremity flexibility compared with the ACG (Table 5). This finding indicated moderate to high effect sizes (η^2^ = 0.114, 0.428, and 0.141). These results are noteworthy because they suggest that immersive virtual reality interventions are effective interventions for older adults, particularly for the parameters of interest. Indeed, the moderate or higher effect sizes support the intervention’s potential efficacy. The literature includes studies reporting the effects of virtual reality interventions on various physical functions. For example, Campo-Prieto et al. conducted a randomized controlled trial involving an immersive virtual reality intervention using the HTC Vive three times a week for 10 weeks in elderly individuals. The results showed positive effects on physical functions such as balance, walking, and hand grip strength compared with traditional interventions, and high levels of satisfaction were reported due to the intervention’s ability to encourage exercise [73]. Hong et al. reported significant improvements in lower extremity muscle strength and balance performance in elderly individuals with degenerative arthritis following an immersive virtual reality intervention compared with a traditional intervention in a randomized controlled study [74]. Considering the two repetitions determined as the Minimal Clinically Important Difference (MCID) limit for the sit-to-stand test in the literature [51], both intervention groups remained below the MCID limits reported in the literature (IVR: 1.77; ACG: 1.09). However, although the MCID value was not exceeded, when interpreted together with the statistically significant improvements observed in the IVR group in the inter-group analyses, the results suggest that the immersive virtual reality intervention had a more significant effect in terms of functional gains. Additionally, Peng et al. emphasized that virtual reality interventions, when combined with traditional interventions, effectively improve physical fitness parameters such as balance, muscle strength, and overall mobility [75]. These results are consistent with the findings of a study reporting that virtual reality interventions promote exercise at higher frequencies and intensities compared with traditional exercise interventions, and that they lead to improvements in physical function outcomes [76]. Additionally, another study found that older adults who participated in virtual reality interventions experienced greater enjoyment and a sense of accomplishment, and developed more positive attitudes toward regular exercise [77]. This could help establish an exercise routine and improve functional assessment outcomes [78]. Although the literature mostly reports lower extremity strength and balance results, the improvements observed in upper extremity strength and lower extremity flexibility in our study, compared with the active control group, provide an additional perspective by demonstrating that immersive virtual reality intervention is also effective on different muscle groups. In conclusion, the significant improvement observed in older adults in the present study compared with the ACG demonstrates not only the effectiveness of a virtual reality-based intervention but also the efficacy of immersive virtual reality technology. Based on the success of IVR interventions on physical functions, the intervention content targeting multiple physical functions, its high level of interaction, task-oriented nature, and enjoyable design can be highlighted as effective factors contributing to improved physical fitness in older adults.

### 4.3. Risk of Falling and Fear of Falling

Although fall risk scores assessed using the MFS showed a decreasing trend in the IVR group, there was no statistically significant difference between the groups when changes in pre-test and post-test scores were compared (Table 6). Consistent with our current findings, Duque et al. reported that virtual reality may be a potential tool, but it alone does not reduce the risk of falls, emphasizing its limitations in fall rehabilitation and prevention [79]. Zahedian-Nasab et al. reported that a virtual reality intervention improved balance performance but, on its own, did not lead to a reduction in fall risk [44]. However, in a randomized controlled study conducted by Parijat et al. on older adults, the virtual reality intervention group was given a slip test on a real slippery surface, followed by treadmill training in a virtual environment with virtual slipping. The control group participated in a similar process by walking normally in a real environment instead of a virtual environment. Both interventions ended with a second slip test. The results showed that the frequency of falls decreased from 50% to 25% in the control group and from 50% to 0% in the virtual reality group [80]. In conclusion, the factors that affect the multidimensional impact of falls in older adults, including physical, psychological, and environmental factors, differences in fall risk assessment methods, intervention content and duration, and sample size, may contribute to differences in study results. In addition, no statistically significant difference was observed in the between-group analyses in terms of fear of falling, as assessed using the FES-I (Table 6). The current finding suggests that the effect of virtual reality interventions on fear of falling may be limited. Virtual reality interventions target inadequate cognitive and motor functions that may cause falls by providing multidimensional activation of individuals’ balance mechanisms through simulations [81,82]. Increasing the cognitive functions of the individual during the intervention, and thereby improving focus and environmental awareness, may contribute to a reduction in the fear of falling. Indeed, a systematic review reported that virtual reality interventions showed promise in improving balance and reducing the fear of falling in older adults [83]. For example, Sánchez et al. suggested that virtual reality interventions may have positive effects on the fear of falling [84], whereas Kwok et al. reported no effect on fear of falling at week 12 but a decrease after 24 weeks in a randomized controlled trial involving a virtual reality intervention [85]. This suggests that the duration of the intervention may impact fall-related effects. Although no significant difference was observed in our study, some factors that may influence the current findings should be taken into consideration. The participants’ good general health and low fear of falling scores at baseline, in accordance with the inclusion criteria, may have limited the potential for measurable improvement following the intervention. It should also be considered that some assessment tools used, such as the FES-I and MFS, may have limited sensitivity for detecting subtle or short-term changes in this population. Although no statistically significant differences in MFS and FES-I scores were observed, the possibility of a ceiling effect cannot be completely ruled out. Another important consideration is the multidimensional nature of fall-related factors in older adults, including physical, psychological, and environmental components. Therefore, virtual reality interventions may be a potential tool for older adults; however, further research is needed to clearly demonstrate their impact on the risk and fear of falls in older adults, including a comprehensive consideration of the multidimensional nature of falls, longer intervention durations, targeted VR interventions, and the use of objective assessment methods.

### 4.4. Fatigue

Comparing the changes in pre-test and post-test scores in terms of fatigue reported using FACIT showed a statistically significant difference between the study groups (Table 7). The decrease in FACIT scores during the post-test was greater in the IVR group than in the ACG. This significant improvement in the IVR group can be attributed to the gamification element, interactive content, motivational properties, and enhanced engagement with the intervention through the multi-sensory inputs of the immersive virtual reality intervention [86,87]. Consistent with our current findings, Gani et al. reported that a virtual reality intervention was effective in reducing fatigue in elderly individuals living in nursing homes [88]. Additionally, Alhusamiah et al. reported that virtual reality interventions in cancer patients resulted in significant improvements in fatigue symptoms [87]. Although the between-group comparisons showed a greater improvement in FACIT scores in the IVR group than in the active control group, the scores of both groups (IVR: 4.27 points; ACG: 2.09 points) remained below the widely cited Minimal Clinically Important Difference (MCID) value for FACIT of 15.9 points that was reported for patients with rheumatoid arthritis [58]. However, despite not exceeding the standard MCID threshold, the statistically significant improvement and greater change in the score of the IVR group relative to that of the ACG suggest that immersive virtual reality interventions may provide promising benefits in alleviating fatigue in relatively healthy older adults. Additionally, the inclusion of rest periods in the design of the immersive virtual reality intervention in our study, taking into consideration the sustainability of the intervention and fatigue levels in older adults, may have also contributed to the effectiveness in terms of fatigue levels.

### 4.5. Quality of Life

The WHOQOL-OLD comprehensively addresses quality of life by including subdimensions such as sensory functions, autonomy, past and future activities, social participation, death and dying, and closeness. Comparison of the final test scores between the IVR group and ACG showed significant differences only in the autonomy and social participation subdimensions, while no significant differences were observed in the other subdimensions or in the total score (Table 8). The current findings support the effectiveness of immersive virtual reality interventions in enhancing autonomy and social participation while indicating that it does not lead to a significant improvement in overall quality of life. Studies have shown that controlled exercises in a safe environment support older adults’ sense of self-efficacy and independence [89,90]. Additionally, virtual reality interventions may expand the domain of social participation by promoting social interactions [91,92,93]. Studies have reported that immersive virtual reality interventions may also be effective in enhancing cognitive health, which is important for maintaining autonomy in daily life [94,95]. In our study, cognitive status represents only one of our inclusion criteria (MMST ≥ 24) (Table 3); however, the findings suggest that cognitive status may influence autonomy in the elderly population, consistent with the existing literature. A systematic review reported that virtual reality interventions are effective in improving memory and overall mental health, as well as enhancing quality of life, in older adults with dementia and mild cognitive impairment [43]. Additionally, Monteagudo et al. reported that physical function in older adults has a greater impact on quality of life than factors such as cognitive function, age, and body mass index [96]. Cacciata et al., in a systematic review, reported that virtual reality interventions did not yield strong results on quality of life due to factors such as sample size, exercise platforms, quality of life assessment tools, intervention duration, and frequency [33]. These findings support the existence of multidimensional factors influencing quality of life. Additionally, in our study, the significantly higher pre-test WHOQOL-OLD total score in the virtual reality intervention group suggests that there may have been a pre-existing difference between the groups at baseline. Therefore, the lack of a significant difference between the groups after the intervention may be associated with the participants’ relatively good general health status and high initial quality of life. Future studies could aim to develop more comprehensive virtual reality interventions targeting all subdimensions of quality of life and overall quality of life with different contents. Moreover, since the WHOQOL-OLD is a self-reported measure, its sensitivity to detecting change may have been limited by subjective factors; however, it remains a widely used and validated instrument in older adult populations.

### 4.6. Treatment Satisfaction

Evaluation of treatment satisfaction in virtual reality and traditional exercise interventions is important for determining the effectiveness and compatibility of interventions. Comparing the treatment satisfaction levels of the groups showed that the IVR group’s treatment satisfaction levels were significantly higher than those of the ACG (Table 9). The mean treatment satisfaction score for the IVR group assessed using the VAS was 9.32 ± 1.09, while that of the control group was 7.45 ± 1.54. Several studies showing that virtual reality interventions can significantly increase satisfaction levels compared with traditional interventions support our findings [97,98]. For example, Dockx et al., in a study involving 281 elderly individuals, found that treatment satisfaction scores assessed from the user’s perspective indicated that the virtual reality intervention was more appealing than traditional interventions [99]. Based on our findings and observations throughout the study, we believe that the interactive nature of immersive virtual reality interventions, their ability to make exercises less routine, and their provision of enjoyable therapeutic experiences contribute to increased treatment satisfaction in older adults.

### 4.7. Virtual Reality Sickness

Virtual reality discomfort is one of the reported side effects of using these technologies. The current findings were quite satisfactory in terms of VRSQ (Table 10). The immersive virtual reality intervention and the equipment used were reliable in terms of measuring virtual reality discomfort in older adults. Proximity–conformity conflict is a factor that may contribute to virtual reality discomfort. It occurs when there is an inconsistency between the distance at which the person’s eyes converge and the distance at which they are aligned [100,101]. The Oculus Quest 2 device used in this study features a mechanism that allows it to be adjusted to suit the person’s eye spacing; furthermore, providing high-resolution screens with a first-person perspective ensures high-quality visuals and reduces the mismatch in eye distance range. These advantages of the device may minimize proximity adaptation conflict and prevent the likelihood of virtual reality discomfort. These findings highlight the importance of integrating virtual reality interventions into exercise plans to ensure that individuals receive the intended benefits without increasing fatigue or discomfort [86,102].

### 4.8. Limitations and Strengths

This study presents a comprehensive approach to the use of an engaging virtual reality application by older adults, a topic that has been less commonly explored in the literature. The study provides a multidimensional impact analysis of physical and psychosocial parameters by creating an active comparison model with a traditional home-based exercise program. The fact that the intervention was conducted in real-life environments outside of a clinical or hospital setting supports the feasibility of integrating virtual reality interventions into the daily lives of older adults. The study’s findings provide important insights for promoting healthy and active aging in the older population and developing feasible interventions in this area. By highlighting the potential impact of virtual reality interventions compared with traditional exercise programs, it serves as a guide for developing effective rehabilitation strategies in the current digital age.

Limitations in sample size may affect the generalizability of the results; however, significant effects may emerge with robust methodologies and comprehensive analyses. In particular, future studies targeting larger samples and more diverse elderly populations could provide important contributions to the literature. While the participants’ demographic characteristics were balanced, it should be noted that there were more female participants, which may limit the generalizability of the findings to the broader older adult population. Additionally, the fact that participants in both groups were relatively healthy and functionally independent at baseline may have limited the potential for observable improvements, particularly in measures such as fall-related parameters and quality of life. Another limitation is the absence of retention data, which prevents the evaluation of the long-term sustainability of the intervention effects. Additionally, the participants resided in different settings; thus, there was a lack of environmental control during the intervention period, and factors such as lighting, noise, or available space may have influenced their engagement and performance. Additionally, the WHOQOL-OLD scale and the FACIT Fatigue Scale used in the study are self-reported measures and may be subject to the participants’ subjective perceptions, which could be considered a potential limitation. The 8-week immersive virtual reality intervention had significant effects on many parameters; however, longer follow-up periods are needed to evaluate long-term effects.

## 5. Conclusions

Compared with traditional home-based exercise interventions, immersive virtual reality interventions are effective in improving physical functions and psychosocial parameters, such as fatigue and quality of life subdimensions (autonomy and social participation), in older adults. Of the technological approaches, immersive virtual reality interventions appear to be a promising tool for the elderly population. Future studies should analyze the cost-effectiveness and real-world applicability of immersive virtual reality interventions in supporting clinical decision-making.

## Figures and Tables

**Table 1:** Sociodemographic characteristics of the participants.

	IVR Group (n = 22)		ACG (n = 22)		Total		t/X^2^	*p*
	n	%	n	%	n	%		
**Gender**								
Female	18	81.82	18	81.82	36	81.82	0.000	1.000
Male	4	18.18	4	18.18	8	18.18		
Age	71.14 ± 4.83		75.36 ± 9.16		73.25 ± 7.55		−1.914	0.062
**Employment Status**								
Not Working	0	0.00	1	4.55	1	2.27	-	-
Retired	1	4.55	5	22.73	6	13.64		
Housewife	16	72.73	15	68.18	31	70.45		
Other	5	22.73	1	4.55	6	13.64		
**Smoking**								
Yes	5	22.73	7	31.82	12	27.27	0.458	0.498
No	17	77.27	15	68.18	32	72.73		
**Alcohol Consumption**								
No	22	100.00	22	100.00	44	100.00		
**Any Illness**								
Yes	16	72.73	16	72.73	32	72.73	0.000	1.000
No	6	27.27	6	27.27	12	27.27		
**Illness**								
Hypertension	14	63.64	12	54.55	26	59.09	-	-
Diabetes	5	22.73	8	36.36	13	29.55	-	-
**Medications**								
Yes	15	68.18	16	72.73	31	70.45	0.109	0.741
No	7	31.82	6	27.27	13	29.55		
**History of Falls**								
No	5	22.73	6	27.27	11	25.00	0.158	0.924
1–2 times	9	40.91	9	40.91	18	40.91		
More than 2	8	36.36	7	31.82	15	34.09		

*p* < 0.05 (chi-square test). IVR: immersive virtual reality; ACG: active control group. Note: Age was analyzed using an independent samples *t*-test; chi-square tests were applied to categorical variables. *p*-values are not reported for employment status due to unmet chi-square test assumptions or for disease status because some participants had multiple diseases.

**Table 2:** Comparison of anthropometric measurements for the IVR group and the ACG.

	Intervention Group	n	$\bar{x}$	s	t	*p*
Height (cm)	IVR	22	161.55	7.95	0.702	0.487
	ACG	22	159.86	7.94		
Weight (kg)	IVR	22	73.77	13.12	0.784	0.437
	ACG	22	70.50	14.53		
Body Mass Index (kg/m^2^)	IVR	22	28.37	5.51	0.544	0.590
	ACG	22	27.52	4.80		

*p* < 0.05 (Independent samples *t*-test).

**Table 3:** Comparison of intra-group and inter-group MMST scores.

	Group	Pre-Test			Post-Test			F	*p*_3_	η^2^
		$\bar{x}$	s	*p*_1_	$\bar{x}$	s	*p*_2_			
MMST	IVR	25.86	1.55	0.866	26.59	1.22	0.239	2.923	0.095	0.067
	ACG	25.95	1.96		26.05	1.76				

*p* < 0.05. MMST: Mini-Mental State Test; IVR: immersive virtual reality; ACG: active control group. *p*_1_: Comparison of pre-test scores between groups (independent samples *t*-test). *p*_2_: Comparison of post-test scores between groups (independent samples *t*-test). *p*_3_: Comparison of changes in pre-test and post-test scores between groups (ANCOVA).

**Table 4:** Comparison of intra-group and inter-group FAB-T total scores.

	Group	Pre-Test			Post-Test			F	*p*_3_	η^2^
		$\bar{x}$	s	*p*_1_	$\bar{x}$	s	*p*_2_			
FAB-T	IVR	28.77	4.68	0.403	34.50	3.81	0.008 *	14.336	0.000 *	0.259
	ACG	27.32	6.59		30.18	6.13				

* *p* < 0.05. FAB-T: Fullerton Advanced Balance Scale; IVR: immersive virtual reality; ACG: active control group. *p*_1_: Comparison of pre-test scores between groups (independent samples *t*-test). *p*_2_: Comparison of post-test scores between groups (independent samples *t*-test). *p*_3_: Comparison of changes in pre-test and post-test scores between groups (ANCOVA).

**Table 5:** Comparison of intra-group and inter-group SFT scores.

	Group	Pre-Test			Post-Test			F	*p*_3_	η^2^
		$\bar{x}$	s	*p*_1_	$\bar{x}$	s	*p*_2_			
Sit-to-stand test	IVR	12.09	3.21	0.147	13.86	2.38	0.010 *	5.301	0.026 *	0.114
	ACG	10.73	2.91		11.82	2.67				
Weightlifting	IVR	14.73	2.21	0.901	17.09	1.60	0.000 *	30.683	0.000 *	0.428
	ACG	14.64	2.59		14.82	1.97				
Two-minute step test	IVR	59.14	9.79	0.533	63.86	10.61	0.822	2.586	0.116	0.059
	ACG	61.55	15.06		63.05	13.27				
Sit-and-reach test	IVR	3.55	7.24	0.084	4.73	7.03	0.032 *	6.733	0.013 *	0.141
	ACG	0.23	4.97		0.50	5.48				
Back scratch test	IVR	−12.95	9.57	0.774	−11.82	8.92	0.600	1.719	0.197	0.040
	ACG	−13.70	7.48		−13.14	7.57				
Eight-step walk test	IVR	5.86	0.90	0.497	5.59	0.92	0.623	0.006	0.937	0.000
	ACG	6.07	1.12		5.74	1.08				

* *p* < 0.05. SFT: Senior Fitness Test; IVR: immersive virtual reality; ACG: active control group. *p*_1_: Comparison of pre-test scores between groups (independent samples *t*-test). *p*_2_: Comparison of post-test scores between groups (independent samples *t*-test). *p*_3_: Comparison of changes in pre-test and post-test scores between groups (ANCOVA).

**Table 6:** Comparison of intra-group and inter-group MFS and FES-I scores.

	Group	Pre-Test			Post-Test			F	*p*_3_	η^2^
		$\bar{x}$	s	*p*_1_	$\bar{x}$	s	*p*_2_			
MFS	IVR	18.18	14.19	0.821	13.64	11.25	0.463	1.692	0.201	0.040
	ACG	17.27	12.22		16.14	11.12				
FES-I	IVR	21.95	5.46	0.486	19.32	5.06	0.106	2.727	0.106	0.062
	ACG	23.41	8.02		21.82	4.98				

*p* < 0.05. MFS: Morse Falls Scale; FES-I: International Fall Efficacy Scale; IVR: immersive virtual reality; ACG: active control group. *p*_1_: Comparison of pre-test scores between groups (independent samples *t*-test). *p*_2_: Comparison of post-test scores between groups (independent samples *t*-test). *p*_3_: Comparison of changes in pre-test and post-test scores between groups (ANCOVA).

**Table 7:** Comparison of intra-group and inter-group FACIT scores.

	Group	Pre-Test			Post-Test			F	*p*_3_	η^2^
		$\bar{x}$	s	*p*_1_	$\bar{x}$	s	*p*_2_			
FACIT	IVR	11.77	9.70	0.689	7.50	5.93	0.072	5.172	0.028 *	0.112
	ACG	12.91	8.99		10.82	6.00				

* *p* < 0.05. FACIT: FACIT Fatigue Scale; IVR: immersive virtual reality; ACG: active control group. *p*_1_: Comparison of pre-test scores between groups (independent samples *t*-test). *p*_2_: Comparison of post-test scores between groups (independent samples *t*-test). *p*_3_: Comparison of changes in pre-test and post-test scores between groups (ANCOVA).

**Table 8:** Comparison of intra-group and inter-group WHOQOL-OLD scores.

	Group	Pre-Test			Post-Test			F	*p*_3_	η^2^
		$\bar{x}$	s	*p*_1_	$\bar{x}$	s	*p*_2_			
Sensory functions	IVR	8.41	1.71	0.125	8.64	1.71	0.073	1.110	0.298	0.026
	ACG	9.32	2.12		9.55	1.57				
Autonomy	IVR	15.95	2.61	0.055	16.68	2.80	0.027 *	2.344	0.133	0.054
	ACG	14.09	3.57		14.18	4.27				
Past, present, and future activities	IVR	15.82	2.15	0.059	16.45	2.24	0.320	0.283	0.598	0.007
	ACG	14.09	3.58		15.45	4.09				
Social participation	IVR	14.68	3.62	0.015 *	15.14	2.38	0.009 *	1.551	0.220	0.036
	ACG	11.91	3.65		12.64	3.58				
Dying and death	IVR	8.55	3.71	0.582	8.41	3.39	0.498	0.162	0.689	0.004
	ACG	9.18	3.89		9.14	3.66				
Closeness	IVR	16.73	2.76	0.305	16.95	2.63	0.958	0.689	0.411	0.017
	ACG	15.82	3.03		16.91	3.05				
WHOQOL-OLD Total	IVR	80.27	8.18	0.039 *	83.86	9.81	0.055	0.896	0.349	0.021
	ACG	74.41	9.97		77.64	11.04				

* *p* < 0.05. WHOQOL-OLD: World Health Organization Quality of Life Instrument—Older Adults Module; IVR: immersive virtual reality; ACG: active control group. *p*_1_: Comparison of pre-test scores between groups (independent samples *t*-test). *p*_2_: Comparison of post-test scores between groups (independent samples *t*-test). *p*_3_: Comparison of changes in pre-test and post-test scores between groups (ANCOVA).

**Table 9:** Comparison of inter-group treatment satisfaction scores.

	Group	n	$\bar{x}$	s	t	*p*
VAS	IVR	22	9.32	1.09	4.649	0.000 *
	ACG	22	7.45	1.54		

* *p* < 0.05 (independent samples *t*-test). VAS: Visual Analog Scale; IVR: immersive virtual reality; ACG: active control group.

**Table 10:** VRSQ scores of participants in the IVR group.

	n	$\bar{x}$	s	Min	Max
VRSQ	22	0.09	0.29	0	1

VRSQ: Virtual Reality Sickness Questionnaire.

## Data Availability

The datasets analyzed in the current study can be obtained from the relevant author upon reasonable request.

## Abbreviations

The following abbreviations are used in this manuscript:

IVR	Immersive Virtual Reality
ACG	Active Control Group
MMST	Mini-Mental State Test
SFT	Senior Fitness Test
FAB-T	Fullerton Advanced Balance Test
MFS	Morse Fall Scale
FES-I	International Fall Efficacy Scale
FACIT	Fatigue Scale
MCDI	Minimal Clinically Important Difference
WHOQOL-OLD	World Health Organization Quality of Life Instrument—Older Adults Module
VAS	Visual Analog Scale
VRSQ	Virtual Reality Sickness Questionnaire
